# Male Rural-to-Urban Migrants and Risky Sexual Behavior: A Cross-Sectional Study in Shanghai, China

**DOI:** 10.3390/ijerph110302846

**Published:** 2014-03-10

**Authors:** Jun-Qing Wu, Ke-Wei Wang, Rui Zhao, Yu-Yan Li, Ying Zhou, Yi-Ran Li, Hong-Lei Ji, Ming Ji

**Affiliations:** 1 School of Public Health, Fudan University, No. 138 Yixueyuan Road, Shanghai 200032, China; E-Mails: wkw168@yeah.net (K.-W.W.); zhaorui821030@163.com (R.Z.); 2 Department of Epidemiology and Social Science on Reproductive Health, Shanghai Institute of Planned Parenthood Research/WHO Collaborating Centre for Research in Human Reproduction Unit of Epidemiology, 2140 XieTu Road, Shanghai 200032, China; E-Mails: lyy1033@163.com (Y.-Y.L.); yingzhou2012@163.com (Y.Z.); llyyyrr@sina.com (Y.-R.L.); hongleiji@gmail.com (H.-L.J.); 3 The Key Laboratory of Family Planning Device of National Population and Family Planning Commission, 2140 XieTu Road, Shanghai 200032, China; 4 College of Nursing University of South Florida, 4202 East Fowler Avenue FAO 100 Tampa, FL 33620, USA; E-Mail: mji@health.usf.edu

**Keywords:** migration, HIV/AIDS, migrants, risky sexual behavior, China

## Abstract

This study examined the prevalence and the determinants of risky sexual behavior (defined as having multiple sex partners and paying for sex) among male rural-to-urban migrants in China. An anonymous questionnaire was used to collect information on socio-demographics, knowledge, attitudes, and behavior associated with increased risk of risky sexual behavior from 4,069 subjects. In total 1,132 (27.8%) participants reported two or more sex partners and 802 (19.7%) participants paid for sex. A considerable proportion (29.6%–41.5%) did not use a condom during risky sexual behavior. Logistic regression analysis revealed that unmarried status (OR: 0.62, CI: 0.42–0.85 for married), earlier age at first sexual experience (OR: 0.67, 95% CI: 0.31–0.91 for ≥22 years old), poor perception of risk of acquiring HIV/AIDS (OR: 1.51, 95% CI: 1.33–1.96 for unlikely; OR: 2.38, 95% CI: 1.61–3.70 for impossible), frequent exposure to pornography (OR: 0.67, 95% CI: 0.60–0.81 for sometimes; OR: 0.31, 95% CI: 0.11–0.43 for never), attitudes toward legalization of commercial sex (OR: 0.39, 95% CI: 0.21–0.59 for no), peer influence (OR: 0.51, 95% CI: 0.27–0.88 for no), and not knowing someone who had/had died from HIV/AIDS (OR: 0.35, 95% CI: 0.20–0.53 for yes) were all significantly associated with having multiple sex partners. Those who paid for sex showed similar findings.

## 1. Introduction

Since the first case of AIDS was reported in 1985 in China, the number of reported HIV infection cases has risen dramatically [1,2]. In 2005, 2007, 2009, and 2011, the total number of HIV infections was 650,000, 700,000, 740,000, and 780,000, respectively, showing a rising trend [1]. The alarming trend has created serious public health problems and has threatened national security, social stability, and economic development in China [2]. In China, about 80% of HIV/AIDS cases are found among rural residents, and three-quarters of the HIV infections are found among people 20–39 years of age [1,3,4,5]. At the same time, the mode of HIV transmission has shifted substantially over time. While, in the past, drug users have represented the largest single cause of HIV transmission in China, the proportion of HIV cases among intravenous drug users decreased from 100% in 1989 to 28.4% in 2011 [1,5]. In 2011, the proportions of HIV infection through heterosexual transmission and homosexual transmission reached 46.5% and 17.4%, respectively [1]. Heterosexual transmission has already become the main mode of transmission in China [1,5,6]. Increased internal migration and increased prevalence of sexually transmitted infections are contributing to this changing pattern of HIV transmission [6]. In addition, HIV infection is quickly spreading from the high-risk groups to the general population in China [1,5,7].

As a special group, rural-to-urban migrants have been thought to have a higher level of sexual risk behavior than rural residents who did not migrate to the cities [8,9]. By the end of 2011, there were an estimated 230 million migrant laborers in China. Nearly half of China’s migrants were born in the 1980s, and the average age is about 28 years [10]. The rural-urban migration has become a significant socioeconomic phenomenon in China. However, owing to the strict Hukou system (household registration system) established in the early 1950s, very few migrants are able to have a nonagricultural status (urban residents), even if they work in an urban city for a long period of time and eventually return to their rural hometown or move to another city [11,12,13,14]. Migrants may act as a bridge population in the spread of HIV as infected migrants return home with the infections and unknowingly pass it on to their sexual partners [15,16,17,18]. In addition, several studies have suggested that rural-to-urban migration may play a crucial role in changing the HIV epidemic in China by widening social and sexual mixing [9,19,20]. Another study found that approximately 70% of HIV infections occur among rural residents, 80% of whom are males [21]. Young, male rural-to-urban migrants have been identified as the tipping point of the AIDS epidemic in China [15].

Condom use is among the most effective means of preventing infection from HIV/AIDS and other sexually transmitted diseases [22]. Consistent and correct condom use is reported to reduce the risk of HIV infection by approximately 69% [23]. However, data from some studies indicate that condom use among male migrants remains extremely limited [20,24,25]. This may explain to some extent how migration can create vulnerability to HIV.

**Figure 1:**
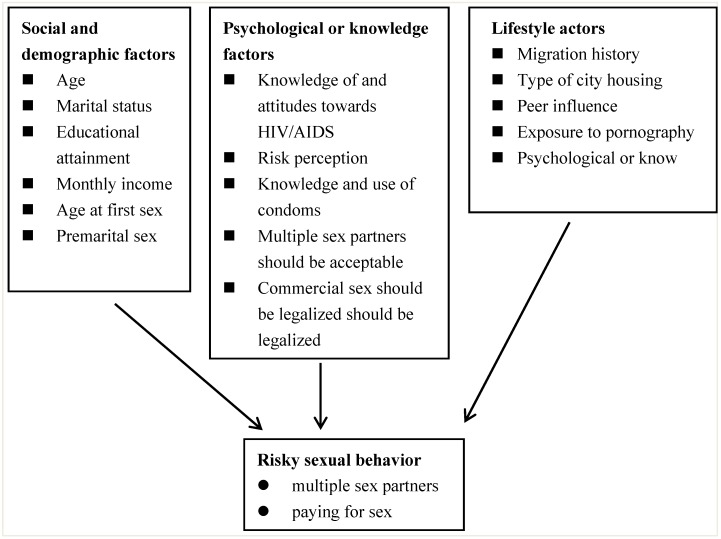
An theoretical framework for the study of male rural-to-urban migrants’ sexual behavior in Shanghai, China.

There have been other explanations for the vulnerability of migrants to HIV/AIDS, such as long periods away from the social controls of family and spouses, low levels of education, limited access to information and services, and alcohol use [9,15,19,24,26]. However, few studies have been conducted to examine the extent of risky sexual behavior and to examine the risk factors for engaging in risky sexual behavior among male migrants in their destination in China. Even fewer studies have further identified the risk factors from the urban environments the migrants work in, which are largely different from their hometowns. For example, Internet bars, prostitution, and “blue” (pornographic) CDs/DVDs/videos, which are rarely found in rural areas, are common in cities. We conducted a survey among male migrants in Shanghai to identify the characteristics of this population, to estimate the prevalence of risky sexual behavior (defined as having multiple sex partners and paying for sex), and to examine the risk factors for engaging in risky sexual behavior.

Our theoretical framework (Figure 1) identifies risky sexual behavior as an outcome variable which is influenced by a range of background factors containing social and demographic, psychological or knowledge and lifestyle factors [27,28,29].

## 2. Methods

### 2.1. Subjects and Procedure

This study only involves subjects who already migrated from rural areas and who are residing in urban areas. The data used in the analysis are from a large and population-based survey conducted in Shanghai, the largest city in China with approximately more than 10 million migrants [30]. The city is administratively divided in to 16 administrative districts and one county. Sample selection followed a two-stage stratified sampling procedure. First, two districts were randomly selected from the city as sources of study participants. Second, the sample subjects were recruited using the quota-sampling procedure from seven different occupational groups (manufacturing, construction, transportation, wholesale and retail trade, accommodations and catering industry, resident services and other services, and health industry). These seven occupational groups were selected because they accounted for approximately 90% of the migrants in each district. The quota-sampling required that the number of subjects recruited from a specific occupational group in the sample be proportional to the number of subjects employed in that specific occupational group in the entire migrant population. Finally, eligibility criteria for selection of migrants included: (1) being a rural-to-urban male migrant; (2) being 18–49 years of age; (3) had resided in the selected districts for at least 12 months; and (4) was sexually experienced. During the fieldwork, interviewers visited the sampled individuals, explained to them the purpose of the study, their right to refuse, and how they would be compensated for their time, and invited them to participate in the study. All interviews were conducted by public health workers who had previous experience in administering epidemiological surveys. The field survey was administered between April 2012 and May 2012, and of the 4,325 migrants who volunteered to participate in the study, 4,069 (94.01%) completed a face-to-face interview, which was undertaken in an environment that was as private as possible and was conducted in Mandarin or in the local dialects, if the respondent could not communicate in Mandarin. It took approximately 35–50 min to complete the questionnaire.

### 2.2. Data Collection

A structured questionnaire was used for the individual interviews. The questionnaire, which was anonymous, requested information about social and demographic characteristics, migration history, knowledge of and attitudes towards HIV/AIDS, risk perception, knowledge and use of condoms, peer influence, exposure to pornography, acquaintance with someone who had or had died from HIV/AIDS and related diseases, and sexual behavior.

### 2.3. Measures

Demographic variables were measured directly from responses to survey questions. The variables were marital status (married and unmarried, including divorced, widowed and single), ethnicity (Han and others), education attainment (elementary school or lower, junior high school, and high school or above), monthly income (<1,000, 1,000–1,999, 2,000–2,999, and 3,000 Yuan or more), total duration of residence in the city (years), and accommodation arrangements (communal dormitory, own room in a dormitory, room with parents, rented room, and other). Although married subjects living with their spouse were likely to engage in less risky sexual behavior, only 14 males migrated and lived with their wives in this study, so they were assigned to various categories of city housing.

For the purposes of this paper, multiple partnerships and commercial sex, which other studies recognized as sexual risk behavior [9,19,26,31], were selected as the dependent variables. These variables were defined as: (1) subjects having had more than one sex partners in the period after migration and (2) subjects having engaged in sexual activity with commercial sex partners during the 12 months preceding the date of the survey. In order to define the variable “having engaging in sexual activity with commercial sex partners” used in this paper, we limited the reference period to the prior 12 months. However, people might not accurately recall the number of sexual partners they had during the period before migration, so we adjusted the reference to indicate the period after the sample subject’s migration. The variable on the number of sexual partners was dichotomized into whether respondents had one or more sexual partners. Participants were asked whether they had paid for sex. Two possible responses (yes/no) were used. At the same time, those who had engaged in risky sexual behavior were asked whether condom use was consistent with their sexual partners (always/sometimes/never), and they were asked if they had used a condom the last time they had sexual intercourse (yes/no). In addition, the respondents were also asked about the age at which they had their first sexual experience.

HIV/AIDS knowledge—the subscale score for HIV/AIDS knowledge (range 0–8)—was created by calculating the correct responses to eight items covering eight questions: three questions on knowledge of HIV/AIDS prevention, five regarding misconceptions about HIV transmission. One point was given for each correct answer, with a possible score ranging from 0 to 8 points. Higher scores indicated a higher level of knowledge. HIV knowledge score was dichotomized classified into <4 as low and ≥4 as high HIV knowledge. For condom knowledge and attitude, subjects were asked whether they thought condom use could effectively prevent HIV/AIDS. Response options were either Yes or No. Risk perception of acquiring HIV/AIDS was categorized into three groups: very possible, unlikely, and impossible. Participant’s attitude toward sexual behavior was evaluated by two items, namely, their agreement with the following statements: (1) “Multiple sex partners should be acceptable”; and (2) “Commercial sex should be legalized” (with the answer options “yes” and “no”).

It is widely accepted that one’s environment exerts profound influences on one’s behavior. When farmers migrated to urban environments greatly different than their hometowns, we cannot be sure whether their sexual behavior was affected by the surrounding environment. Therefore, we initially explored a few potential risk factors for engaging in risky sexual behavior due to migration, such as the total duration of residence in the city (years), type of city housing, exposure to pornography, and peer influences, in addition to the conventional factors of demographic characteristics, awareness, attitude, and knowledge explored in other studies [9,15,19,24,26].

Peer influence was defined as whether a migrant had peers who had more sexual partners or who engaged in commercial sex. In this study, migrants’ peers refer only to friends and workmates with whom they were acquainted after migrating to city. In addition, the respondents who had paid for sex were asked the following questions: (1) “Did you go out with peers when you had your first commercial sex experience?” and (2) “Did you go out with peers the last time you engaged in commercial sexual intercourse?” For exposure to pornography, respondents answered separately whether they frequently, sometimes, or never viewed pornography. Those who had ever been exposed to pornography were further asked: (1) “What types of pornography do you primarily read or watch (movies, videos, novels, magazines, or pictures)?” and (2) “Where do you mainly watch pornography (Internet, CDs/DVDs, mobile telephone, magazines, or other venue)?” The attitudes towards person with HIV/AIDS were evaluated by following five items: (1) “Once your friend was infected HIV, you would keep a normal friendship with him/her?”; (2) “HIV-infected people should be dismissed?”; (3) “Whether or not willing to live with person with HIV/AIDS?”; (4) “Whether or not willing to shake hands with an individual infected with HIV”; and (5) “Whether or not willing to go to dinner with person with HIV/AIDS?” These measures used were similar to those employed in other studies on exposure to pornography and perceptions of HIV infection [19,24,32,33,34].

### 2.4. Statistical Analysis

Data on all the questionnaires were entered using double entry by different professionals using EpiData 3.1 in order to enable a comparison between the data and correction of data entry mistakes. Analysis was conducted using SAS version 9.2 (SAS Inc., Cary, NC, USA). A chi-square test was performed to test the relationships between potential risk factors and outcomes. Multivariate logistic regression analyses were carried out to determine the risk factors for participants’ risky sexual behavior. All the independent variables that were statistically significant in the univariate analyses were step-wisely selected with a criterion of *p* < 0.05 for entry and of *p* < 0.10 for removal. Odds ratios (ORs) and 95% CIs were used as indictors of the strength of association. *p* < 0.05 was considered statistically significant.

### 2.5. Ethics

This study was approved by the Ethics Committee of Fudan University. Written consent was obtained from all respondents before the interview.

## 3. Results

### 3.1. Sample Characteristics

Table 1 presents the selected background characteristics of the sample respondents. Of minority groups. The mean age of the participants was 30.1 years with a range of 18–49 years, and about half (51.6%) of the participants were under 30 years. Approximately half (48.2%) were married. Over half (54.7%) had completed at least a junior high school education. More than half (52.5%) had resided in the city for at least 7 years. About two-fifths (38.9%) of the participants lived in a collective dormitory with others and 29.7% of the respondents had their own room in a dormitory.

### 3.2. HIV/AIDS-Related Knowledge

Though most of the participants (88.7%) correctly knew the major three routes of HIV transmission, misunderstandings in relation to HIV transmission weren’t uncommon among the respondents. Nearly half (49.2%) of participants believed that HIV can be contracted through mosquito bites, and 12.1% thought a person could be infected from dining with an HIV positive person. All the subjects thought a woman infected with HIV should not conceive a child, and 90.3% thought that HIV can be transmitted from a pregnant woman to her child. Only 45.1% of participants thought that consistent condom use could prevent HIV transmission (data not shown), but approximately one-quarter (23.7%) thought that regular use of antibiotics could prevent HIV transmission (Table 2).

**Table 1:** Demographic characteristics of migrants (*n* = 4,069).

Variable	*n*	%
Ethnicity		
Han	3,809	93.6
Other	260	6.4
Age (years)		
<20	407	10.0
20–29	1,691	41.6
30–39	1,470	36.1
≥40	501	12.3
Mean age (mean)	30.1	
Marital status		
Unmarried	2,106	51.8
Married	1,963	48.2
Educational attainment		
Elementary school or lower	416	10.2
Junior high school	2,227	54.7
High school or more	1,426	35.1
The total duration of residence in the city (years)		
<4	1,058	26.0
4–7	876	21.5
7–10	809	19.9
≥10	1,326	32.6
Monthly income (Yuan)		
<1,000	710	17.4
1,000–2,000	1,452	35.7
2,000–3,000	1,327	32.6
≥3,000	580	14.3
Type of city housing		
Collective dormitory with others	1,584	38.9
Own room in a dormitory	1,210	29.7
Room with parents	802	19.7
Room rented with others	307	7.5
Other	166	4.1

### 3.3. HIV/AIDS-Related Attitudes and Stigma

Overall, the participants had a negative attitude toward people with HIV/AIDS, as reflected in this study, where over one-quarter (28.3%) thought that they would end a friendship with a friend with HIV/AIDS and about two-fifths (41.9%) thought people with HIV/AIDS should be dismissed from work. More half (56.6%) would feel ashamed if they had to live with HIV/AIDS, about one-quarter (26.4%) were unwilling to shake hands with an individual infected with HIV, and 39.3% were unwilling to go to dinner with people with HIV/AIDS (data not shown).

**Table 2:** HIV/AIDS-related knowledge among migrants (*n* = 4,069).

Knowledge	Number of Correct Responses	%
Blood/blood product transmission	3,585	87.9
Mother-to-child transmission	3,674	90.3
Sexual transmission	3,601	87.9
Eating together	492	12.1
Insect bite	2,002	49.2
Antibiotic protection	964	23.7
Hugging and shaking hands	1,457	35.8
Kiss	2,071	50.9

### 3.4. Sexual Behavior

Of the 4,069 respondents used in this analysis, most (97.3%) reported having sexual intercourse during the last 12 months and the mean age at the time of first intercourse was 20.2 years. A total of 328 (8.1%) reported engaging in oral sex with their partners in the last 12 months, and only 19 (0.5%) participants reported having anal sexual intercourse with their partners. About half (47.3%) reported premarital sex. In our study, 1,132 (27.8%) participants reported having two or more sexual partners (with the maximum number of partners being 26), and 802 (19.7%) participants reported having sex with a commercial sex partner at least once during the last 12 months (Table 3). About one-fifth (216) of two or more sex partners who are actually commercial sexual partners.

After univariate analyses of the factors associated with risky sexual behavior, multiple sex partners among migrants showed significant associations with older age, unmarried status, earlier age at first sexual experience, premarital sex, longer total duration of residence in the city (years), poor perception of acquiring HIV/AIDS, frequent exposure to pornography, lower HIV/AIDS-related knowledge score, acceptability of multiple sex partners, attitude toward legalization of commercial sex, having peers who had engaged in risky sexual behavior, and not knowing someone who had/had died from HIV/AIDS. Those who paid for sex showed similar findings, with the following differences: total duration of residence in the city (years), the perception of acquiring HIV/AIDS and HIV/AIDS-related knowledge score weren’t significantly associated with migrants’ commercial sexual behavior, while monthly income (Yuan) was significantly associated with it (Table 4).

### 3.5. Condom Use

All the respondents who had engaged in risky sexual behavior were asked about their use of condoms in their lifetime and during their last sexual encounter. Of the 1,132 respondents with multiple partners, just 281 (24.8%) reported always using a condom with their sexual partners in their lifetime, while 53.5% never used a condom (Table 5). Among the 802 respondents who had intercourse while engaging in paid sex only 35.4% reported that they always used a condom, whereas 29.6% reported that they had never used a condom during intercourse (Table 5). Condom use during the last sexual encounter was also quite low among the respondents (Table 6). Only one-third (33.9%) of participants with multiple sexual partners, and about three-fifths (58.5%) of the respondents with commercial sex partners, reported condom use with their sexual partners (Table 6).

**Table 3:** Sexual behavior among migrants (*n* = 4,069).

Variable	*n*	%
Sexual intercourse during the last 12 months		
Yes	3,959	97.3
No	110	2.7
Age at first sex		
<22	2,356	57.9
≥22	1,713	42.1
Oral sexual intercourse		
Yes	328	8.1
No	3,741	91.9
Anal sexual intercourse		
Yes	19	0.5
No	4,050	99.5
Premarital sex		
Yes	1,925	47.3
No	2,144	52.7
Number of sexual partners in one’s lifetime		
1	2,937	72.2
≥2	1,132	27.8
Had sex with sex workers		
Yes	802	19.7
No	3,267	80.3

### 3.6. Peer Influence and Pornography

Of those who had paid for sex, approximately three-fifths (59.5%) went out with peers when they had their first commercial sex experience while 72.3% went out alone the last time they had commercial sexual intercourse. The proportion of those who had been exposed to pornography by watching movies and videos, reading novels and magazines, or viewing pictures was 78.7%, 56.3%, 18.5%, 30.5% and 28.1%, respectively. The Internet (48.3%) was the main source for the participants who were exposed to pornography, followed by CDs/DVDs (36.6%), mobile telephones (11.8%), and magazines (2.1%) (data not shown).

**Table 4:** Univariate analyses of factors associated with risky sexual behavior.

Variables	Multiple Sexual Partners				Paying Sex			
	≥2%	OR	95% CI	*p*	Yes %	OR	95% CI	*p*
Age (years)								
<20	74 (18.2)	1		<0.001	49 (12.0)	1		<0.001
20–29	455 (26.9)	1.7	1.53–1.87		361 (21.3)	2.0	1.39–3.21	
30–39	441 (30.0)	1.9	1.26–2.71		307 (20.9)	1.9	1.38–3.14	
≥40	162 (32.3)	2.1	2.27–2.73		85 (17.0)	1.5	1.11–1.94	
Marital status								
Unmarried	795 (37.7)	1		<0.001	517 (24.4)	1		0.001
Married	337 (17.2)	0.34	0.15–0.67		285 (14.5)	0.52	0.26–0.89	
Educational attainment								
Elementary school or lower	112 (26.9)	1		0.19	101 (24.3)	1		0.084
Junior high school	599 (26.9)	0.99	0.53–1.51		463 (20.8)	0.81	0.40–1.25	
High school or above	421 (29.5)	1.13	0.60–1.60		338 (23.7)	0.95	0.59–1.38	
Monthly income (Yuan)								
<1,000	171 (24.1)	1		0.069	113 (15.7)	1		<0.001
1,000–2,000	405 (27.9)	1.22	0.86–1.72		266 (18.3)	1.19	1.06–1.35	
2,000–3,000	381 (28.7)	1.27	0.90–1.71		274 (20.7)	1.40	1.15–1.69	
≥3,000	175 (30.2)	1.36	1.02–1.80		149 (25.7)	1.85	1.38–2.35	
Type of city housing								
Collective dormitory with others	447 (28.2)	1		0.111	324 (20.4)	1		0.104
Own room in a dormitory	352 (29.1)	1.04	0.93–1.20		251 (20.7)	1.01	0.54–1.53	
Room with parents	194 (24.2)	0.81	0.65–0.97		143 (17.8)	0.77	0.33–1.25	
Room rented with others	93 (30.3)	1.11	0.97–1.26		54 (17.5)	0.75	0.30–1.21	
Other	46 (27.7)	0.98	0.87–1.10		30 (18.1)	0.85	0.40–1.39	
The total duration of residence in the city (years)								
<4	253 (23.9)	1		0.002	183 (17.2)	1		0.081
4–7	254 (28.7)	1.29	1.06–1.59		171 (19.5)	1.16	0.92–1.46	
7–10	257 (31.8)	1.48	1.21–1.82		177 (21.8)	1.34	1.06–1.69	
≥10	368 (27.6)	1.22	1.02–1.47		271 (20.5)	1.23	0.99–1.51	
Age at first sex								
<22	767 (32.5)	1		<0.001	491 (20.8)	1		0.034
≥22	365 (21.4)	0.57	0.49–0.65		311 (18.1)	0.84	0.72–0.99	
Premarital sex								
Yes	599 (31.1)	1		<0.001	404 (21.3)	1		0.014
No	533 (24.9)	0.73	0.63–0.81		398 (18.2)	0.82	0.76–0.96	
Risk perception of acquiring HIV/AIDS								
Very possible	37 (14.6)			<0.001	46 (18.3)	1		0.112
Unlikely	242 (16.0)	1.10	1.02–1.19		275 (18.2)	0.99	0.86–1.16	
Impossible	853 (37.1)	3.42	1.13–3.75		481 (20.8)	1.18	0.99–1.41	
Exposure to pornography								
Frequently	493 (33.2)	1		<0.001	436 (32.2)	1		<0.001
Sometimes	322 (25.9)	0.70	0.59–0.83		245 (14.3)	0.35	0.19–0.50	
Never	318 (23.7)	0. 63	0.52–0.74		121 (12.0)	0.28	0.11–1.47	
HIV/AIDS-related knowledge								
<4	446 (30.0)	1		0.022	315 (21.2)	1		0.068
≥4	686 (26.6)	0.84	0.74–0.98		487 (18.8)	0.86	0.74–1.01	
Multiple sex partners should be acceptable								
Yes	484 (37.6)	1		<0.001	315 (24.5)	1		<0.001
No	648 (23.3)	0.51	0.44–0.58		487 (17.5)	0.27	0.23–0.32	
Commercial sex should be legalized								
Yes	421 (30.6)	1		0.005	297 (21.6)	1		<0.001
No	711 (26.4)	0.81	0.71–0.94		505 (18.8)	0.84	0.71–0.99	
Condom use could prevent HIV/AIDS								
Yes	517 (29.3)	1		0.072	373 (21.1)	1		0.43
No	615 (26.7)	0.88	0.77–1.01		429 (18.5)	0.85	0.73–0.99	
Whether or not have peers who had engaged in risky sexual behavior								
Yes	752 (38.3)	1		<0.001	427 (21.7)			0.001
No	380 (17.8)	0.35	0.31–0.41		375 (17.7)	0.78	0.67–0.91	
Whether or not knew someone who had or had died from HIV/AIDS								
No	501 (39.2)	1		<0.001	385 (30.5)	1		<0.001
Yes	631 (22.6)	0.43	0.37–0.50		417 (14.8)	0.39	0.34–0.47	

**Table 5:** Participants’ use of condoms with sexual partners.

	Multiple Sexual Partners		Paying Sex	
	*n*	%	*n*	%
Always used	281	24.8	284	35.4
Sometimes used	245	21.7	281	35.0
Never used	606	53.5	237	29.6

**Table 6:** Participants’ use of condoms at the last sexual encounter.

	Multiple Sexual Partners		Paying Sex	
	*n*	%	*n*	%
Used condom	384	33.9	469	58.5
Did not use condom	748	66.1	333	41.5

### 3.7. Multivariate Results

To investigate further, logistic regression analysis was carried out to identify the factors associated with risky sexual behavior (Table 7). After controlling for potential confounding variables, having multiple sexual partners was significantly associated with the following covariates: marital status, age at first sexual experience, perception of acquiring HIV/AIDS, exposure to pornography, attitude regarding commercial sex, peer influence, and whether or not they knew someone who had/had died from HIV/AIDS.

Another dependent variable, having sex with commercial sex partners was significantly associated with marital status, exposure to pornography, attitude towards commercial sex, peer influence, and whether or not they knew someone who had/had died from HIV/AIDS.

**Table 7:** Logistic regression analysis of factors correlated with ever having risk sexual behavior.

Variable	Multiple Sex Partners			Paying Sex		
	OR	95% CI	*p*	OR	95% CI	*p*
Marital status						
Unmarried	1		<0.001	1		0.033
Married	0.62	0.42–0.85		0.77	0.60–0.95	
Age at first sex						
<22	1		0.035	1		0.056
≥22	0.67	0.31–0.91		0.86	0.72–1.03	
Risk perception of acquiring HIV/AIDS						
Very possible	1		<0.001	1		0.39
Unlikely	1.66	1.51–1.75		0.98	0.85–1.17	
Impossible	2.42	2.27–2.62		1.2	0.93–1.52	
Exposure to pornography						
Frequently	1		0.007	1		0.001
Sometimes	0.67	0.60–0.81		0.55	0.28–0.83	
Never	0.31	0.11–0.43		0.26	0.11–0.42	
Commercial sex should be legalized						
Yes	1		0.005	1		0.001
No	0.39	0.21–0.59		0.41	0.29–0.55	
Whether or not have peers who had engaged in risky sexual behavior						
Yes	1		<0.001	1		<0.001
No	0.51	0.27–0.88		0.17	0.10–0.26	
Whether or not knew someone who had or had died from HIV/AIDS						
No	1		<0.001	1		<0.001
Yes	0.35	0.20–0.53		0.41	0.24–0.59	

Marital status was a strong protective association with having more than one sex partner and engaging in sexual activity with commercial sex partners. Compared with the unmarried participants, the married participants were 0.62-fold less likely to have two or more sexual partners and 0.77-fold less likely to purchase for sex, respectively.

Respondents who were ≥22 years old the first time they had sex were less likely (37%) to have multiple sexual partners compared to those whose first sexual experience occurred at an earlier age (<22 years old). We also found a significant inverse relationship between perception of acquiring HIV/AIDS and having multiple sexual partners, while no significant association between risk perception and purchasing sex was found. Those who perceived themselves to be at a very possible risk were 2.42-times more likely to have multiple sexual partners than those who did not do so.

Compared to those who frequently viewed pornography, the respondents who never viewed pornography were 69% less likely to report multiple sexual partners and 74% less likely to pay for sex. Attitude toward legalization of commercial sex increased the likelihood of transitioning to both having multiple sexual partners (1/0.39 times) and having sex with sex workers (1/0.41 times).

Peer influence was a significant factor in engaging in risky sexual behavior. Respondents who had peers that had one or more sexual partners or who engaged in commercial sex were 1.96-times (1/0.51) and 5.88-times (1/0.17) more likely, respectively, to have multiple sexual partners and to pay for sex than those who did not have peers having one or more sexual partners or engaging in commercial sex.

Last, knowing someone who had/had died from HIV/AIDS also demonstrated a strong protective association with having multiple sexual partners and paying for sex. Those who had known someone had died of AIDS were 0.35- and 0.41-times less as likely to have multiple sexual partners and pay for sex, compared with those who had never known someone who had died from AIDS, respectively.

## 4. Discussion

The current study documented higher rates of risky sexual behavior among migrants in the place of destination, including having more than one sex partner and engaging in sexual activity with commercial sex partners, and several findings are consistent with previous research studies conducted in China [24,27,34] and in other countries [35,36]. Unmarried males were more likely to have commercial sex than married males. A similar finding has also been reported in an earlier study [37]. An alternative explanation is that sexually active individuals pay for sex in order to meet their physical and psychological demands and to avoid feeling lonely. Our study results also suggest that premarital sex was prevalent among the study participants, especially in the younger group and the unmarried participants. This phenomenon may result from the fact that, in this study, more of the unmarried participants were younger males, not older males.

An alarming finding from this study was that condom use was quite low among the migrants who engaged in risky sexual behavior. A considerable proportion (29.6%–66.1%) of the male migrants was also found not to use a condom during risky sexual behavior and only 45.1% of the migrants knew condom use could reduce the risk of becoming infected with HIV. A similar result is reported in a previous study [35]. These results show that sexually active individuals did not routinely practice safe sex, leading to a high risk for HIV infection. The low rate of condom use may be the result of poor knowledge about the protective effects of condoms against HIV. Another explanation may be that there is a lack of access to condoms and they may feel embarrassed to carry condoms or purchase them in front of others. The low awareness and use of condoms has important implications in HIV infection and transmission.

At the same time, HIV/AIDS-related knowledge was poor among migrants. Misinformation about HIV transmission and prevention was especially widespread. About half of the participants believed that mosquitoes carried HIV and one-quarter thought that HIV could be prevented by regular use of antibiotics. Insufficient HIV/AIDS-related knowledge can promote migrants’ risky sexual behavior [35]. The perception of infection with HIV/AIDS among migrants was low. In this study, 20.8%–37.1% of the respondents thought that it was impossible for them to be infected with HIV, despite engaging in many risky sexual relationships; some even never used a condom during sexual relations. Stigma was not uncommon; for example, a fair number of migrants were unwilling to make friends with an individual infected with HIV or to eat dinner with people who have HIV/AIDS. This stigma may be one of the major barriers that could prevent the success of intervention programs conducted by governments.

Multiple logistic analyses identified the factors associated with having multiple sexual partners. In agreement with the findings of previous research more information such as the author and year of publication [38], this study found a negative relationship between risk perception and risky sexual behavior. A possible explanation for this result is that migrants have lower levels of education, and they are especially lacking in HIV/AIDS-related knowledge. In this study, despite the finding that the age at which the respondents engaged in their first sexual experience hasn’t decreased significantly, compared to the mean age of 20 years at first sexual intercourse in another study [24], logistic analysis found that the age a person was at his first sexual experience was significantly associated with having multiple partners, but not associated with purchasing sex. This finding is consistent with previous studies [24,39] having regarded first sexual intercourse at an earlier age as a potential risk behavior.

A surprising result in this study is that males are particularly likely to report risky sexual behavior when they viewed pornography. Although it is not known whether the relationship between exposure to pornography and sexual behavior is causative, these data suggest that it is urgent to improve migrants’ awareness about the seriousness of exposure to pornography, which can increase the likelihood of involvement in risky sexual partnerships. Attitude toward legalization of commercial sex was not only significant in relation to having multiple partners; it was also significant in relation to purchasing sex. Similar results in previous studies in China and elsewhere [24,34] have also shown that the permissive attitude towards risky sexual behavior increases the likelihood of engaging in unprotected sex. The results of this current study lend support to this finding.

A noteworthy finding in this current study is that peer influence showed a significant independent association with having multiple sex partners and purchasing sex. This result concurs with the result of another study [27]. It is also worth noting that substantial proportions (59.5%) of respondents who engaged in paid sex indulged in their first commercial sexual behavior with others, while 72.3% engaged in their last commercial sexual encounter on their own. This implies that peer influence occupies an important role in increasing the likelihood of involvement in risky sexual behavior among migrants. This might be explained by social influence from peers, which was confirmed to exert significant influence over an individual’s HIV risk behavior. However, we do not know if the relationship between peer influence and sexual behavior is a causal link in this cross-sectional study. A longitudinal study should be conducted to further explore this association.

A protective factor of sexual risk behavior among migrants is that migrants have known someone who has been infected with HIV or who has died from AIDS. This finding is corroborated by other studies [40]. Severe symptoms, such as chills, fever, cough, body aches, weakness, significant weight loss, and skin herpes among those who have been infected with HIV or died from AIDS, likely act as warnings to migrants. More importantly, the transmission route among people infected with HIV might teach migrants that unprotected sexual behavior is a risk factor for HIV acquisition and transmission.

In logistic models, educational level, monthly income (Yuan), and type of city housing were not significantly associated with risky sexual behavior. However, several studies [33,38,41] have indicated that low income, low levels of education, and poor living conditions may lead to higher-risk behavior among migrants. On the contrary, other studies [24,34,36] have shown no link between risky sexual behavior and formal education, monthly income, and type of city housing. The present study supports the latter conclusion. These associations should be explored further.

These findings support the need for HIV prevention interventions and control among male migrants. First, these findings suggest that intervention programs need to be implemented among migrants, with the aim of increasing safe-sex practices, condom use, appropriate messaging, and services targeting these vulnerable populations. Second, given the high mobility of migrants, it is more practical to set up comprehensive education programs about HIV/AIDS-related knowledge, awareness of personal vulnerability to HIV infection, and reduction of risk behavior targeting migrants, especially for rural residents before they leave for cities to pursue a temporary job. Third, the implication is that HIV intervention programs need to extend beyond individual risk-takers to also target their broader social network of peers. Fourth, action is needed to dispel the stigmas associated with the disease and to reduce misconceptions about HIV transmission. Lastly, more intense efforts should be made to improve unmarried males’ awareness about the seriousness of exposure to pornography and to help them develop a good attitude toward pornography.

In this study, several limitations should be acknowledged. First, due to the private, intimate and sensitive nature of sexual behavior, it is possible to underestimate of the prevalence of risk sexual behavior. This may result in doubts about the reliability of reported sexual behavior. Second, the cross-sectional nature of the data erodes our ability to establish causal associations. Third, our findings may be subject to recall bias.

## 5. Conclusions

The present study contributes to the growing literature on high-risk sexual behavior among migrants in their place of destination. The findings document that a higher proportion of migrants are engaging in HIV high-risk behavior. Our study also identifies possible risk factors for engaging in risky sexual behavior among migrants, such as unmarried status, earlier age at first sexual experience, poor perception of acquiring HIV/AIDS, frequent exposure to pornography, attitude toward legalization of commercial sex, having peers who had engaged in risky sexual behavior, and not knowing someone who had/had died from HIV/AIDS. More importantly, two strong risk factors were found: exposure to pornography and peer influence associated with having multiple sex partners and purchasing sex. To our knowledge, few studies have been conducted to examine the effects of pornography and peer influence on male migrants’ risky sexual behavior in China. There is therefore the need to focus policy and program attention on male migrants in China, particularly those engaging in risky sexual behavior.
